# Investigation of the potentiation of the analgesic effects of fentanyl by ketamine in humans: a double-blinded, randomised, placebo controlled, crossover study of experimental pain[ISRCTN83088383]

**DOI:** 10.1186/1471-2253-5-2

**Published:** 2005-04-02

**Authors:** Adam P Tucker, Yong Ik Kim, Raymond Nadeson, Colin S Goodchild

**Affiliations:** 1 Department of Anaesthesia and Perioperative Medicine, Monash Medical Centre, 246 Clayton Road, Melbourne, Victoria 3168, Australia; 2 Department of Anesthesiology, Soonchunhyang University Hospital, Seoul, Korea; 3 Department of Anaesthesia Monash University, Melbourne, Australia

## Abstract

**Background:**

Despite preclinical evidence suggesting a synergistic interaction between ketamine and opioids promoting analgesia, several clinical trials have not identified dosing regimens capable of eliciting a benefit in the co-administration of ketamine with opioids.

**Methods:**

Ten healthy volunteers participated in a double blinded, randomised, placebo controlled, crossover laboratory study in order to determine whether a low dose of ketamine potentiated the antinociceptive effect of fentanyl without causing an increase in sedative effects. A battery of tests was used to assess both nociception and sedation including electrical current, pressure, thermal stimuli, psychometric tests, and both subjective and objective scores of sedation. Target controlled infusions of the study drugs were used. Ketamine and fentanyl were administered alone and in combination in a double-blinded randomised crossover design. Saline was used as the control, and propofol was used to validate the tests of sedation. Cardiovascular and respiratory parameters were also assessed.

**Results:**

The electrical current pain threshold dose response curve of fentanyl combined with ketamine was markedly steeper than the dose response curve of fentanyl alone. While a ketamine serum concentration of 30 ng/ml did not result in a change in electrical pain threshold when administered alone, when it was added to fentanyl, the combination resulted in greater increase in pain threshold than that of fentanyl administered alone. When nociception was assessed using heat and pressure stimuli, ketamine did not potentiate the anti-nociceptive effect of fentanyl. There was no difference between the sedative effect of fentanyl and fentanyl in combination with ketamine as assessed by both subjective and objective measures of sedation. Cardiovascular and respiratory parameters were unaffected by the study drugs at the doses given.

**Conclusion:**

A serum concentration of ketamine that did not alter indices of sedation potentiated the antinociceptive effect of fentanyl. This potentiation of antinociception occurred without an increase in sedation suggesting that low steady doses of ketamine (30–120 ng/ml) might be combined with μ opioid agonists to improve their analgesic effect in a clinical setting. (296 words)

## Background

Ketamine was patented in 1966 [1], and has long been known to be associated with short-term analgesia [2]. Considerable interest was renewed in ketamine with the discovery that it could block the NMDA receptor and therefore it has a potential role in the management of *windup *and prevention of subsequent spinal cord sensitisation. To date, clinical trials that have investigated its use as an analgesic drug have often described its adverse effects. This has led some authors to question its use in the management of postoperative pain [3].

Several animal studies have suggested that the mechanisms for a synergistic interaction between ketamine and opioids might exist [4] and [5,6]. that combinations of opioids and NMDA receptor antagonists might result in an enhanced effect [7] – as might be predicted by the different mechanisms of action of these classes of drugs [8,9].

The current investigation explored the interaction between ketamine and the opioid fentanyl in the anticipation that a low dose of ketamine might potentiate the analgesic effect of fentanyl. Furthermore, it was hypothesised that the interaction of these drugs might be associated with selective potentiation of analgesia without associated increased sedation; that is that potentiation might occur in the context of a very low dose of ketamine that was not otherwise associated with brain effects such as sedation. It was hoped that the identification of such doses of ketamine may enable better future management of both opioid sensitive physiological pain and NMDA receptor mediated sensitisation without the disadvantage of increased sedation.

## Methods

This study was conducted using a double blinded, randomised, placebo controlled, crossover methodology to determine whether a low dose of ketamine potentiated the antinociceptive effect of fentanyl without potentiating the sedative effect of fentanyl. A battery of tests was assembled to assess both nociception and sedation. Tests of nociception used electrical current, pressure, and thermal stimuli. Sedation was assessed by a subjective and objective score in addition to psychometric tests. Saline was used as the control and propofol was used to validate the tests of sedation. Cardiovascular and respiratory parameters were also monitored in order to detect the occurrence of adverse events.

This investigation was approved by the Southern Health Human Research and Ethics Committee (Project number 96022A and 97074A) in accordance with the guidelines of the National Health and Medical Research Council, Australia (NHMRC). Ten healthy male volunteers were recruited via bulletin board advertisements. The volunteers were trained in the test procedures employed and medically screened. Volunteers were excluded if they had a history of cardiac, neurological, or musculoskeletal disease. Other exclusion criteria included a history of drug abuse, pain syndromes, myasthenia gravis, acute narrow angle glaucoma, asthma, or heart failure, concurrent use of any analgesics, sedatives, erythromycin, MAO inhibitors, or allergy to propofol, fentanyl, or ketamine.

The ten volunteers each attended five three-hour laboratory sessions on separate occasions. In each session, the volunteer received either one of the drug treatments or saline (Table 1). Therefore, each volunteer was exposed to each of the five treatments, over five sessions, with the order of treatment randomised for each volunteer. During each session, the test battery was performed prior to drug administration as a measure of 'baseline' and then repeated when each of the four targeted concentrations were reached.

**Table 1:** Drug Concentrations Targeted

	Baseline	Concentration 1	Concentration 2	Concentration 3	Concentration 4
Placebo (saline)	-	-	-	-	-
Propofol (μg/ml)	0.00	0.15	0.30	0.60	0.90
Ketamine (ng/ml)	0.00	15.00	30.00	60.00	120.00
Fentanyl (ng/ml)	0.00	0.20	0.40	0.80	1.20

Ketamine (ng/ml) &	0.00	30.00	30.00	30.00	30.00
Fentanyl (ng/ml)	0.00	0.20	0.40	0.80	1.20

The orders of tests within the test battery were not varied. The physiological measures were conducted first, followed by the sedation tests, and lastly the nociception tests. Before and after each test battery was performed, a blood sample was taken to establish the drug serum concentration. The duration of each test battery was approximately 20–30 minutes, and between each battery the volunteer was instructed to rest for approximately 20 minutes while the drug serum concentration was increased according to the administration protocol.

The drugs were administered by serum target controlled intravenous infusions (Stanpump; Shafer, CA. 94304, USA, revision November 5, 1996; and a Harvard 22 syringe pump). This method was utilised to maintain a stable serum concentration for the duration of the test battery. Two identical computer and syringe pump systems using opaque intravenous tubing were employed and operated in parallel at all times. The syringe pumps were hidden from both the investigator and the volunteer within an opaque sound-proofed box. Two intravenous catheters (20–22 gauge) were inserted – one for the infusion of the study drug and the other for the withdrawal of blood for serum concentration assay.

Blood samples were transferred to a SST gel and clot activator vacutainer (Becton Dickinson, Franklin Lakes, NJ, USA) and allowed to clot. Immediately after clotting, the samples were centrifuged at 3500 rpm for 10 minutes. The serum was then frozen in liquid nitrogen before being stored in a refrigerator – fentanyl and ketamine samples were stored at -4°C whereas propofol samples were refrigerated at -20°C. A scientist who was blinded to the serum concentrations targeted conducted the analysis of the serum samples. Propofol was analysed using a method similar to Plummer [10]. Fentanyl and ketamine concentrations were analysed using a method based on that of Bjorkman and Stanski. [11].

The pain threshold to electrical current was determined using a computer controlled constant current stimulator (Amlab International Pty Ltd, NSW 2113, Australia). A train of five 1 ms unipolar rectangular pulses, at a frequency of 200 Hz, lasting 25 ms was delivered using single-use disposable Silver-silver chloride electrodes (9013S0241, Medtronic Dantec, NSW, Australia) with a contact area of 0.54 cm^2 ^applied 2 cm apart to the medial non-dominant wrist. The increasing and decreasing staircase method was used to determine the electrical pain threshold, which was defined as the minimum amount of current resulting in a stimulation that was graded as "painful". The mean of three consecutive measurements was used in subsequent analysis.

The pain threshold to contact heat was determined using the ascending ramp method (Somedic Thermotest, Somedic AB, Sweden; applied to the volunteer's non-dominant wrist). A contact thermistor (Hewlett Packard patient monitor M1165A, Model 54S Mass. 02254, USA) was used to monitor the temperature of the volunteer's skin (adjacent to the wrist on the non-dominant forearm) during the session. The pain threshold to pressure was measured using the ascending ramp method (Somedic Algometer, Somedic, Sweden; applied to the non-dominant middle-finger nail bed). Pressure was applied at the increasing rate of 40 kPa/s over a contact surface area of 1 cm^2^. For both tests, the mean of five consecutive measurements was used in subsequent analysis.

The volunteers were asked to rate the symptom of sedation with a visual analogue score by placing a vertical mark through a horizontal 100 mm line with a pencil. The caption "I feel drowsy" was printed above the horizontal line and the left and right ends of the line were labelled with the statements, "not at all" and "extremely" respectively. The investigator – who was unaware of the nature of the drug treatment – assessed the volunteers level of sedation using the Observer Assessment of Alertness/Sedation Scale (OASS) [12]. These scores were measured once during each test battery.

The Symbol Digit Modalities Test (SDMT) is a pen and paper test in which nine symbols are paired with digits and 110 blank squares associated with digits are required to be filled in within 90 seconds [13]. Parallel forms were used in random order. The number of errors that were made during the SDMT test was recorded and an incidental recall task immediately following the primary test was performed. For this, the volunteers were given a new sheet composed of a line of 15 symbols in which all nine symbols were included at least once. The volunteer was then asked to fill in the number associated with the symbol. Where a symbol appeared more than once, and the volunteer correctly identified the number on one occasion and incorrectly on another occasion, credit for the correct identification was given.

A simple auditory reaction time was measured by computer (Amlab International Pty Ltd, NSW 2113, Australia). In this test, the volunteer pushed a micro-switch button in response to a tone using a handset in the dominant hand. The Finger Tapping test was included to assess the effect of the study drugs on the motor nervous system. The volunteers were asked to tap a micro-switch contained within a modified computer serial mouse (Microsoft Corporation, USA) as rapidly as possible over ten seconds and the number of taps were counted. For both of these tests the mean of five consecutive measurements was used in the subsequent analysis.

During each test battery a Hewlett Packard patient monitor (M1165A, Model 54S, Mass. 02254, USA) was used to measure the volunteers' blood pressure, pulse rate, and pulse oximetry. In addition, a small-volume circuit consisting of a mouthpiece, Wrights Respirometer, and an in-line Capnometer sensor (Hewlett Packard, Model 14360A, Mass. 02254, USA) was used to determine the respiratory rate, tidal volume (averaged from ten consecutive breaths), and the end-tidal carbon dioxide. The minute volume was calculated from the tidal volume and the respiratory rate.

The results were analysed by repeated-measures analysis of variance (ANOVA), incorporating the Greenhouse-Geisser adjustment for multisample asphericity (compound asymmetry). For each outcome variable the analysis was conducted across the five treatments (placebo, propofol, ketamine, fentanyl, and fentanyl and ketamine combined), and over the five ascending doses. Within the ANOVA, the hypothesis tested was that provided by the within-subject interaction term *Drug by Dose*. The result of testing this factor was that this method tested for parallelism of the response curves across *ascending doses *according to *drug treatment*. Thus, it identified differences between the drugs with respect to the slope or profile of each dose response curve. Because the above hypothesis was tested for each of the 18 outcome measures subjected to ANOVA, the familywise Type I error-rate was controlled by the Ryan-Holm step-down Bonferroni procedure. A value of *P *≤ 0.05 was regarded as statistically significant. Further analysis and illustration was confined to those dose response curves that were significantly different between the study drugs. When a significant difference between the dose response curves was determined, the dose response curves were ranked according to similarity in order to establish the origin of the significant difference. This was achieved by a stepwise addition of each dose response curve until a significant difference was detected by ANOVA testing.

The OAAS scores were analysed by Friedman's test because of the lack of variance of the OAAS scores at baseline. The profile of the response curve of each volunteer to the increasing drug concentrations was summarised by the sum of the OAAS scores across the five concentrations of each drug. The summary of each drug profile was then blocked by the term *volunteer*. Where a significant difference between the drug profiles was detected, the profiles were classified according to similarity in order to establish the origin of the significant difference. This was achieved by a stepwise addition of profile data with similar appearances until a significant difference was detected by the Friedman test in a similar manner as previously described with ANOVA. Statistical calculations were conducted using Minitab (Release 13.31) for the Friedman Test and SPSS for Windows (Release 10.0.7) for all other procedures.

## Results

Ten volunteers completed all of the experiments and none were excluded or withdrawn. The mean age, weight, and height (standard deviation) of the group were 23 (4.2) years, 66.3 (4.6) kg, and 173.5 (33.5) cm. The study drugs had a dose-dependent effect on the electrical current and pressure pain, SDMT, subjective sedation VAS and OASS, reaction time, and finger-tapping tests (Table 2 and Table 3). The remaining tests did not demonstrate a difference between the study drugs and placebo.

**Table 2:** Differences between the Profiles of the Study Drug Dose Response Curves

Outcome Variable	*P *value	
*Nociception Tests*		
Electrical pain threshold	<0.01	*
Pressure pain threshold	<0.01	*
Heat pain threshold	0.99	
Skin temperature	0.59	

*Sedation Score*		
Subjective sedation visual analogue score	0.01	*
OASS	<0.01	*

*Psychometric Tests*		
SDMT	0.03	*
SDMT errors	0.17	
SDMT recall	0.99	
Reaction time	<0.01	*
Finger tapping test	<0.01	*

*Physiological Tests*		
Systolic blood pressure	0.34	
Diastolic blood pressure	0.99	
Heart rate	0.99	
Respiratory rate	0.99	
Tidal volume	0.73	
Pulse oximetry	0.99	
End tidal carbon dioxide	0.99	
Minute volume	0.99	

SDMT denotes symbol digit modalities test; OASS, observer assessment of alertness/sedation scale; *, *P *≤ 0.05

**Table 3:** Origins of the Significant Differences between the Study Drugs with Respect to the Profile of the Dose Response Curves (P ≤ 0.05)

Outcome Variable	Origins of Difference in Profile by Drug
*Nociception Tests*	
Electrical pain threshold	fentanyl & ketamine versus ketamine, fentanyl versus saline
Pressure pain threshold	fentanyl & ketamine, fentanyl, versus ketamine, saline

*Sedation Score*	
Sedation visual analogue	ketamine, fentanyl, propofol, fentanyl & ketamine versus saline
OASS	propofol versus ketamine, fentanyl, fentanyl & ketamine, versus saline

*Psychometric Tests*	
SDMT	propofol versus ketamine, fentanyl, fentanyl & ketamine versus saline
Reaction time	propofol versus ketamine, fentanyl, fentanyl & ketamine, saline
Finger tapping test	propofol versus ketamine, fentanyl, fentanyl & ketamine, saline

SDMT denotes symbol digit modalities test; OASS, observer assessment of alertness/sedation scale.

**Figure 1:**
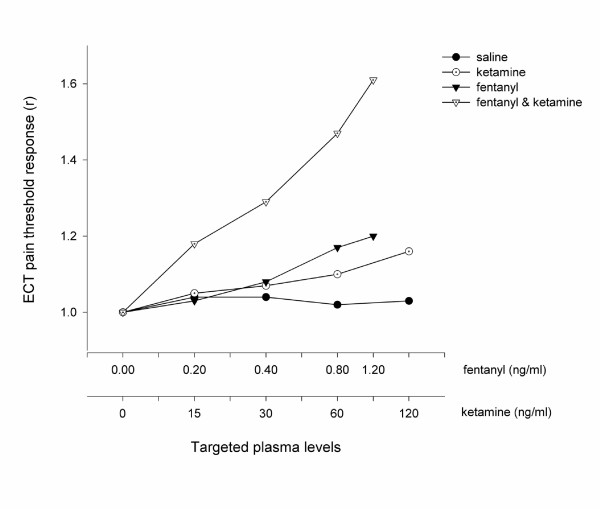
The Antinociceptive Effect Measured using ECT. The electrical pain threshold is expressed as the mean electrical pain threshold of all volunteers (*n *= 10) when a steady serum concentration of the treatment drug had been achieved. These values are standardised by the threshold value at baseline in order to illustrate the difference in profiles between each treatment arm. The serum concentrations targeted are listed in Table 1 and enumerated on the abscissa using a logarithmic scale.

All drugs resulted in a dose dependent increase in the electrical pain threshold compared with placebo (Figure 1). The dose response curve of fentanyl combined with ketamine was markedly steeper than the dose response curves of fentanyl alone (*P *< 0.05; ANOVA). While a ketamine serum concentration of 30 ng/ml did not result in a change in electrical pain threshold when administered alone (*P *= 0.32; two-way ANOVA), when it was added to a fentanyl serum concentration of 0.4 ng/ml the combination resulted in greater increase in pain threshold than that of fentanyl administered alone (*P *< 0.01; two-way ANOVA).

**Figure 2:**
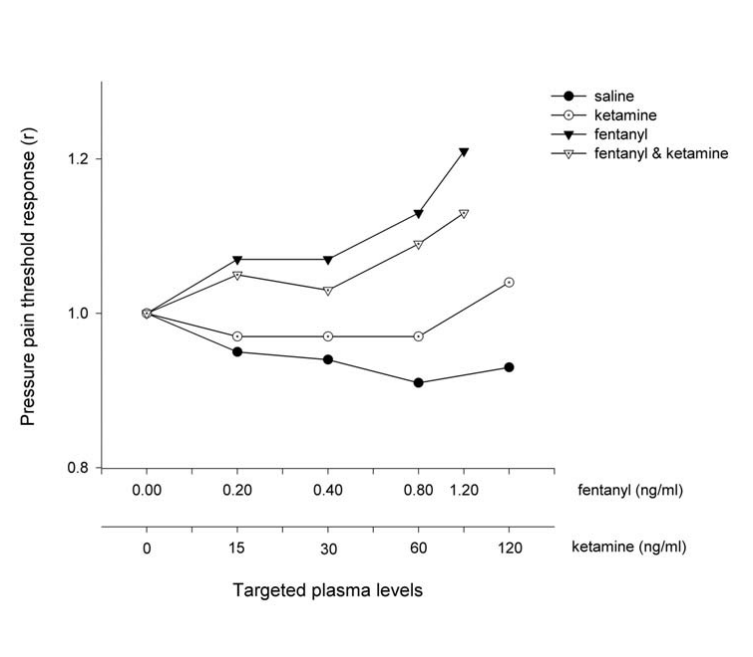
The Antinociceptive Effect Measured using Pressure Algometry. The pressure pain threshold was expressed as the mean pressure pain threshold of all volunteers (*n *= 10) when a steady plasma level of the treatment drug had been achieved. These values were then standardised by the threshold value at baseline in order to illustrate the difference in profiles between each treatment arm. The serum concentrations targeted are listed in Table 1 and enumerated on the abscissa using a logarithmic scale.

**Figure 3:**
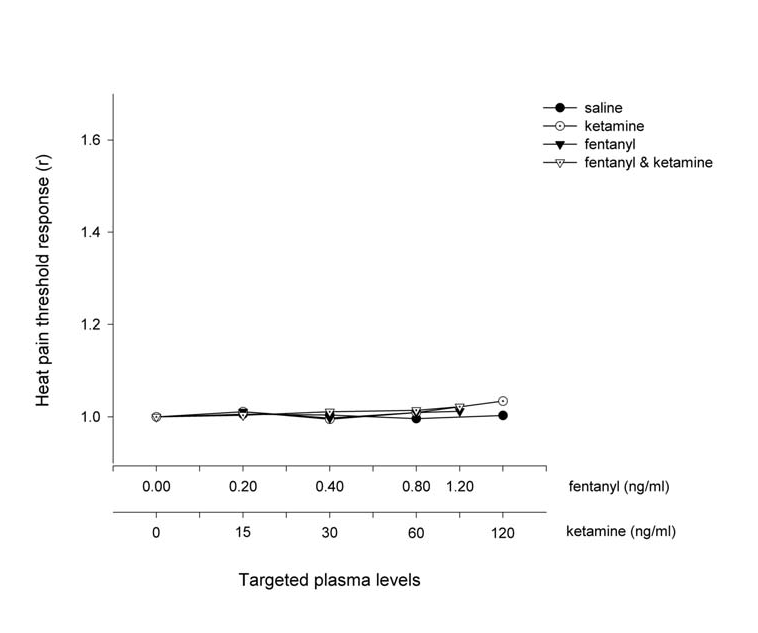
The Antinociceptive Effect Measured using Heat. The heat pain threshold was expressed as the mean heat pain threshold of all volunteers (*n *= 10) when a steady plasma level of the treatment drug had been achieved. These values were then standardised by the threshold value at baseline in order to illustrate the difference in profiles between each treatment arm. The serum concentrations targeted are listed in Table 1 and enumerated on the abscissa using a logarithmic scale.

Fentanyl, both alone and in combination with ketamine, produced a dose dependent increase in pressure pain threshold compared with saline, whereas ketamine alone was ineffective at all doses (Figure 2). The dose response profiles of fentanyl alone and in combination with ketamine were not different (*P *= 0.35; ANOVA). No difference was seen between the study drugs (including placebo) when assessed with heat (Figure 3).

**Figure 4:**
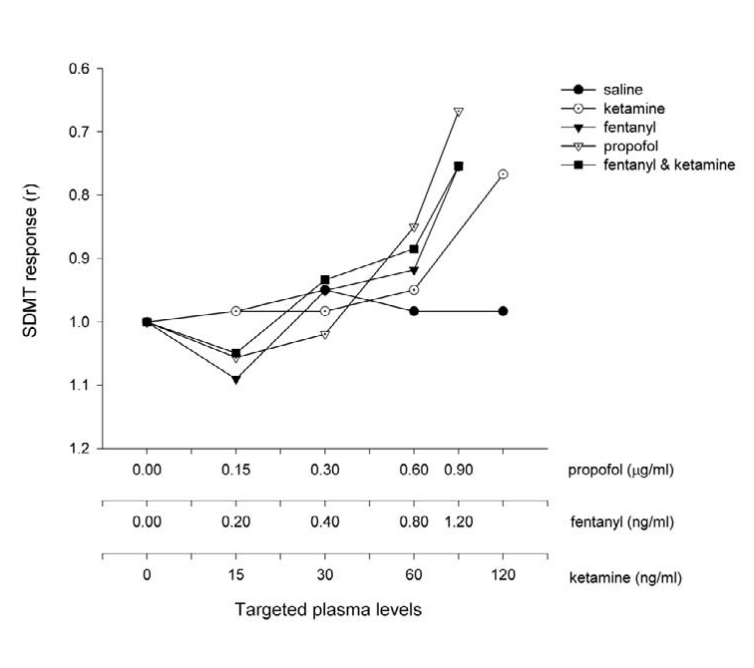
The Psychometric Effect Measured by SDMT The SDMT. score was expressed as the mean score of all volunteers (*n *= 10) when a steady serum concentration of the treatment drug had been achieved. These values are standardised by the threshold value at baseline in order to illustrate the difference in profiles between each treatment arm. The serum concentrations targeted are listed in Table 1 and enumerated on the abscissa using a logarithmic scale.

**Figure 5:**
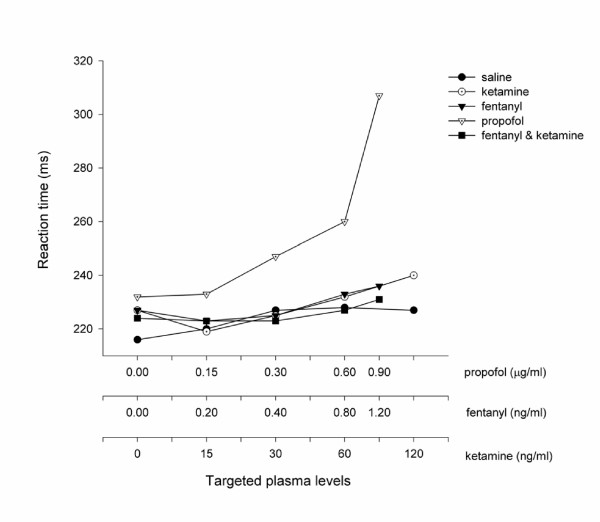
Psychometric Effect Measured by Reaction Time. The reaction time was expressed as the mean of all volunteers (*n *= 10) when a steady serum concentration of the treatment drug had been achieved. These values are standardised by the threshold value at baseline in order to illustrate the difference in profiles between each treatment arm. The serum concentrations targeted are listed in Table 1 and enumerated on the abscissa using a logarithmic scale.

**Figure 6:**
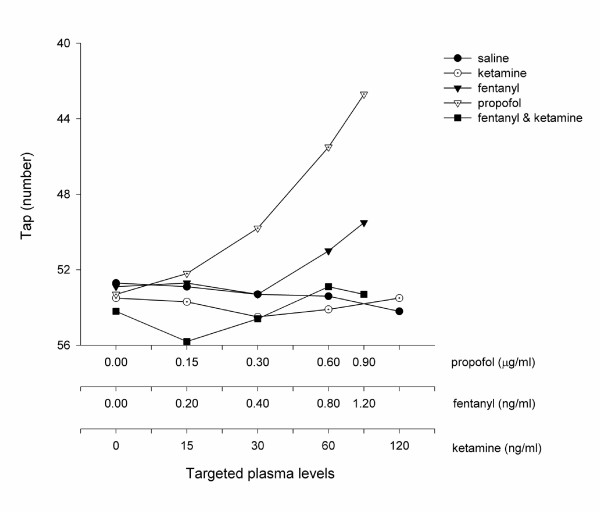
Psychometric Effect Measured by the Finger Tapping Test. The number of finger taps was expressed as the mean of all volunteers (*n *= 10) when a steady serum concentration of the treatment drug had been achieved. These values are standardised by the threshold value at baseline in order to illustrate the difference in profiles between each treatment arm. The serum concentrations targeted are listed in Table 1 and enumerated on the abscissa using a logarithmic scale.

**Figure 7:**
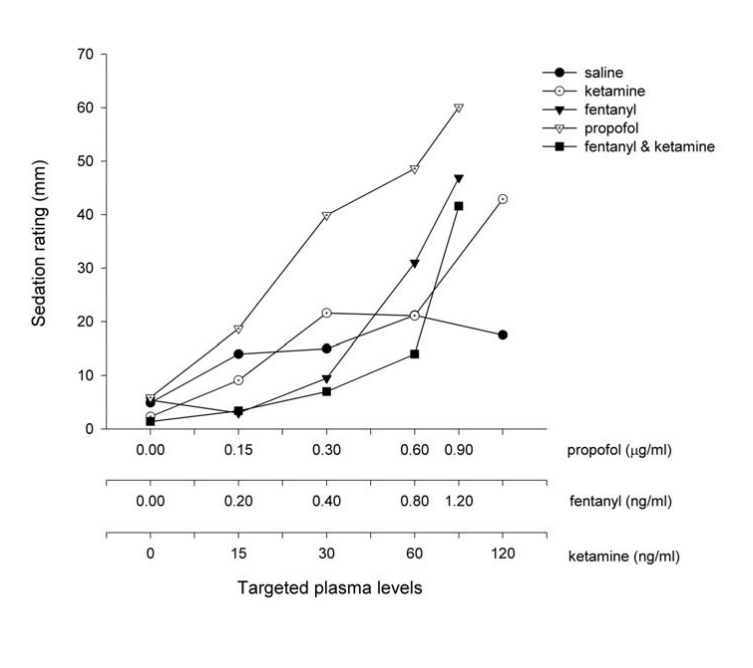
Subjective Sedation Measured by Visual Analogue Score. This figure shows the effect of the five treatments on the visual analogue score (VAS) given to the statement, "I feel drowsy". The VAS was expressed as the mean score of all volunteers (*n *= 10) when a steady serum concentration of the treatment drug had been achieved. All drug treatments were associated an increase in VAS compared to saline. The serum concentrations targeted are listed in Table 1 and enumerated on the abscissa using a logarithmic scale.

**Figure 8:**
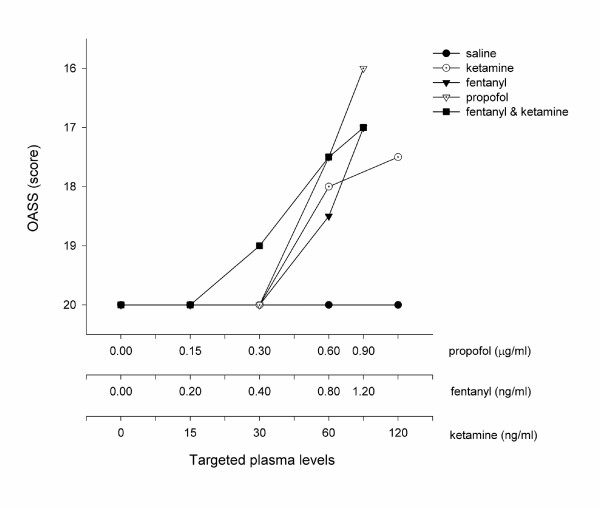
Sedation Measured by OASS. The OASS was expressed as the median score of all volunteers (*n *= 10) when a steady serum concentration of the treatment drug had been achieved. The serum concentrations targeted are listed in Table 1 and enumerated on the abscissa using a logarithmic scale.

All of the study drugs were associated with increased sedation compared with placebo (Table 1) when assessed by the objective psychometric tests (Figure 4, 5, and 6) and the scores of sedation (Figure 7 and 8). Propofol was associated with a more marked subjective sedative effect than either fentanyl or ketamine – alone or in combination (Figure 7**Error! Reference source not found.**). There was no difference between the sedative effect of fentanyl and fentanyl in combination with ketamine as assessed by the subjective sedation VAS, OASS, and SDMT (Figure 4**Error! Reference source not found.**). Specifically, fentanyl 0.4 ng/ml combined with ketamine 30 ng/ml was not associated with increased sedation in comparison with fentanyl 0.4 ng/ml alone. While Propofol was associated with a reduced number of finger taps and an increase in reaction time compared with placebo, none of the other study drugs had any significant effect on these measures of sedation.

The serum concentrations remained steady at each concentration targeted and the ketamine serum concentration remained steady throughout the duration of the experiment when it was combined with fentanyl in a fixed dose (Table 4).

**Table 4:** Measured Serum Drug Concentrations

	Baseline		Concentration 1		Concentration 2		Concentration 3		Concentration 4	
	Mean	SD	Mean	SD	Mean	SD	Mean	SD	Mean	SD
Propofol (μg/ml)	0.00	(0.00)	0.10	(0.04)	0.26	(0.07)	0.58	(0.18)	0.92	(0.24)
			0.11	(0.03)	0.31	(0.11)	0.62	(0.16)	0.91	(0.25)

Ketamine (ng/ml)	0.00	(0.00)	11.49	(4.31)	24.64	(6.68)	47.87	(18.03)	85.75	(31.87)
			12.61	(4.85)	27.48	(11.62)	51.86	(21.54)	93.88	(38.26)

Fentanyl (ng/ml)	0.00	(0.00)	0.28	(0.03)	0.53	(0.09)	1.01	(0.15)	1.48	(0.26)
			0.31	(0.07)	0.55	(0.13)	1.02	(0.21)	1.53	(0.25)

Ketamine (ng/ml)	0.00	(0.00)	25.59	(13.26)	30.29	(13.94)	30.73	(16.98)	29.88	(12.84)
			25.41	(5.76)	35.41	(15.90)	29.82	(12.26)	31.58	(14.23)
&										
Fentanyl (ng/ml)	0.00	(0.00)	0.27	(0.11)	0.49	(0.13)	1.05	(0.17)	1.57	(0.26)
			0.29	(0.06)	0.57	(0.15)	1.09	(0.24)	1.57	(0.29)

SD denotes standard deviation. The upper and lower data in each cell represents the serum concentration measured at the beginning and at the end of each test battery respectively.

## Discussion

Pre-clinical investigation of the antinociceptive effect of ketamine and morphine by Chapman and Dickenson [8] led Schmid, Sandler and Katz [14] to hypothesize that there may be a dose of ketamine that has no analgesic potency on its own, but when used in combination with an opioid might produce superior pain relief than either drug alone. To test this hypothesis, the present study constructed a dose response curve for ketamine, from which a dose of ketamine that had no antinociceptive effect was identified (30 ng/ml). When this dose of ketamine was combined with fentanyl the resulting antinociception, as assessed by electrical current pain threshold, was greater than that of either drug alone and clear potentiation was demonstrated. In addition, while ketamine potentiated the antinociceptive effect of fentanyl, the sedative effect was not increased. Therefore, the data presented here supports the hypothesis put forward by Schmid *et al *[14].

A review of the recent preclinical work investigating the interaction between opioid agonists and NMDA antagonists shows mixed results. Some authors have demonstrated that NMDA antagonists enhance the antinociceptive effect of opioids [15,16]. whereas others have been unable to confirm these findings [17,18].

Some authors such as Redwine *et al *[19] have gone so far as to suggest that NMDA receptors are not involved in acute opiate mediated analgesia. In order to resolve these difficulties, a number of investigators have suggested that factors other than receptor interactions may have resulted in the heterogeneous results seen. These factors may include; the opioid agonist and the NMDA antagonist chosen for investigation [19-23], the nociceptive test chosen [16], and the species used for investigation [24].

The findings of this current investigation support the hypothesis that the nociceptive test used is an important determinant of the outcome of investigations assessing the interaction between ketamine and fentanyl, that is, the interaction between ketamine and fentanyl is stimulus dependent. Despite fentanyl-mediated analgesia being clearly potentiated by ketamine when assessed by ECT, this was not seen when it was assessed by pressure algometry. The inability of pressure to demonstrate potentiation was consistent with other investigators who have found that the antinociceptive activity of ketamine on pressure nociception is dependent on preconditioning, such as preconditioning which produces neuropathic pain [25]. Clearly such preconditioning was absent in the current study.

This study encountered similar difficulties to that of Sethna *et al *who found that a large variation in the assessment of C-fibre mediated pain made capsaicin – and in our case heat – a difficult modality to use in the study of nociception [26]. The global failure of all of the drug infusions to increase the heat pain thresholds suggests that either the assessment of heat pain threshold was inadequate or the drug doses used were too low. The heat pain thresholds were assessed by the ascending ramp method with a continuous stimulus, which increased intensity at a constant rate. This method was more susceptible to provoking conditioned timed responses in the volunteers compared with the discrete stimuli used for the assessment of electrical current pain thresholds. Other factors that weakened the assessment of heat pain thresholds included the mechanical stimulation caused by contact of the thermode, which may produce Aβ-fibre mediated interference of the C-fibre sensory input. The authors hypothesize that the use of discrete heat stimuli – such as that produced by laser [27] – may have improved heat stimulation's ability to demonstrate the nature of the interaction between fentanyl and ketamine using this paradigm.

Clinical studies assessing the interaction of ketamine and opioids have also produced mixed results however, consistent with the findings of the current study, a review of the interaction between ketamine and opioids in alleviating clinical pain has demonstrated that ketamine enhances opioid-mediated analgesia [28]. Schmid *et al *in their review of the clinical use of ketamine for the management of postoperative pain differentiated between a high and a low dose range of ketamine [14]. Both the sedative and the psychotomimetic effects of ketamine are known to be dose dependent [29]. As opposed to idiosyncratic drug effects, the dose responsive nature of the unwanted sedative and psychotomimetic effects of ketamine provided the opportunity that a low dose of ketamine could be used to provide analgesia without their occurrence. However, due to the large overlap in the respective ketamine dose response curves for analgesia and sedation, separation of these effects was difficult when ketamine was used alone. Clinical studies using ketamine alone for analgesia have often reported adequate analgesia with associated psychotomimetic effects, or have reported an acceptable level of adverse effects but poor analgesia [30-33].

The primary advantage of combinational therapy relies on the improvement of the desired effect without a concomitant increase in its adverse effects, which some authors have seen as a requirement for the demonstration of a synergistic interaction [26]. That is, it is considered that a synergistic interaction exists between two drugs when the effect of a combination is greater than the sum of the effects produced by each drug given alone. However, it is clear that whilst an increase of the desired effects is desirable, what is critical for clinical advantage is that the occurrence of adverse effects is not increased in a similar manner. Some authors consider that an increase in the beneficial effects of a combination without an increase in the adverse effects is more important than the existence of synergy *per se *[34].

Human dose ranging studies of ketamine are scarce and consequently the doses and methods of administration of ketamine in clinical studies are numerous. Schmid *et al *proposed that dose ranging studies were required as part of a research program to explore the clinical usefulness of ketamine as an adjunct to opioid analgesia in 1999 [14]. In this study, the highest serum concentrations of ketamine targeted were related to regimens that have been used for analgesia clinically [35-37]. While the human laboratory paradigm is one step removed from the clinical arena, it is well suited to dose ranging studies that are vital for the rational planning of clinical investigations.

Clinical and human laboratory studies investigating ketamine for the management of postoperative pain are heterogenous in design and varied in their appraisal of the effect of ketamine in conjunction with opioids for the management of pain [38-52]. In these trials ketamine has been given by the epidural, intramuscular, and intravenous routes, in addition to the description of intrathecal use [53-59]. Javery *et al *described reduced pain ratings and morphine consumption following microdiscectomy when patients received a combination of ketamine and morphine rather than morphine alone [44]. By contrast most studies have investigated pain following abdominal surgery and the results have been less encouraging [45,46]. While these studies may yet lead to the conclusion that the surgical procedure conducted is important in determining whether ketamine augments postoperative opioid analgesia or not; other factors such as the dosing regimen also varies across these studies and appears to be a dominant reason for such variable results.

Studies that have combined ketamine with morphine in an intravenous Patient Controlled Analgesia (PCA) pump have usually failed to demonstrate a reduction in morphine consumption [45-48]. This may reflect the mismatch of ketamine's and morphine's respective time response curves for analgesia. Compared with morphine, ketamine is a short acting drug. Ketamine's short duration of action was demonstrated when it reduced ischaemic arm pain for less than 5 and 10 minutes respectively when used intravenously in doses of 125 μg/kg and 250 μg/kg [2]. Therefore, if the use of the PCA pump is predominantly determined by the longer acting morphine, the combination of ketamine and morphine may result in troughs of ketamine's serum concentration, and occasional peaks when the PCA pump is triggered. This is consistent with the finding that ketamine co-administered with morphine in PCA pumps did not improve patients' VAS in six studies overall when assessed by weighted mean difference [28]. Therefore, because ketamine has a small therapeutic window within which analgesia is not accompanied by adverse effects, great importance should be placed on the dosing regimen chosen.

Some studies have used steady intravenous infusions of ketamine in addition to PCA pump morphine to relieve abdominal postoperative-pain. Both manual infusions and computer controlled serum targeted infusions of ketamine have been studied [38-41,49-52]. However, while it may be expected that a steady manual infusion should result in a stable serum concentration of ketamine, this has not always been shown to be the case. Owen *et al *studied a regimen of ketamine consisting of a bolus and an infusion which lasted 24 hours [39]. The subsequent analysis of the serum concentrations showed that a constant serum concentration was achieved in fewer than half of the thirty patients studied and that in the majority of patients the serum concentrations rose continuously. Therefore, while the published literature describing the use of ketamine suggests that the dose of ketamine needs to be carefully controlled, a manual infusion may not always achieve the level of stability required. As dosage is important in avoiding ketamine's side effects and ensuring adequate NMDA receptor antagonism, the potentiation of analgesia resulting from the combination of ketamine with fentanyl that was found in this study is likely to require a similar attention to dosing. In keeping with this suggestion, Adriaenssens *et al *have shown improved pain ratings and decreased morphine consumption following laparotomy by targeting a ketamine serum concentration of 100 ng/ml using the same pharmacokinetic software used in this study [38]. This present study suggests that ketamine may provide enhanced opioid analgesia at a third of this dose without adverse effects. Notwithstanding this, in contrast to ketamine administered in PCA pumps, ketamine co-administered with morphine infusions have been shown to improve patients' VAS in seven studies overall when assessed by weighted mean difference [28].

An advantage of human laboratory investigation is the ability to use greater number of drug doses and combinations than is usually practical in clinical investigations. There are few barriers to applying the knowledge gained from this study to present day clinical medicine. Doses and serum concentrations of ketamine have been identified using a freely available pharmacokinetic program and using the racemic form of ketamine, which is licensed for clinical use internationally. The findings of this study and those from published clinical studies suggest that accurate and consistent administration of low doses of ketamine are required in order to enhance the antinociceptive effect of opioids and avoid adverse effects.

## Conclusion

The effect of intravenous ketamine and fentanyl was studied in human volunteers using nociceptive and sedative tests. A serum concentration of ketamine that did not alter indices of sedation potentiated the antinociceptive effect of fentanyl. This potentiation of antinociception occurred without an increase in sedation suggesting that low doses of ketamine might be combined with μ opioid agonists to improve their analgesic effect in a clinical setting.

## Abbreviations

ANOVA analysis of variance

MAO Monoamine oxidase

NHMRC National Health and Medical Research Council, Australia

NMDA N-methyl-D-aspartate

OASS Observers Assessment of Alertness and Sedation score

PCA patient controlled analgesia

SD standard deviation

SDMT Symbol Digit Modalities test

SPSS statistical package for the social sciences

VAS visual analogue score

## Competing interests

The authors have not received reimbursements, fees, funding, or salary from an organisation that may in any way gain or lose financially from this publication, nor is such an organisation financing the article-processing charge for this article. The authors do not hold stocks or shares in an organisation that may gain or lose financially from the publication of this paper.

## Authors' contributions

AT, RN, and CG conceived of the study. AT designed and coordinated the study, custom-built equipment and wrote the software, conducted the experimental sessions, performed the data analysis, and wrote the manuscript. YIK assisted with the conduct of the study. All authors read and approved the final manuscript.

## Pre-publication history

The pre-publication history for this paper can be accessed here:


